# Understanding the impact of professional motivation on the workforce crisis in medicine: a rapid review

**DOI:** 10.3399/BJGPO.2021.0005

**Published:** 2021-03-31

**Authors:** Efioanwan Andah, Blessing Essang, Charlotte Friend, Sarah Greenley, Kathryn Harvey, Maria Spears, Joanne Reeve

**Affiliations:** 1 Academy of Primary Care, Hull York Medical School, University of Hull, Hull, UK

**Keywords:** workforce, job satisfaction, retention, NHS, general practice

## Abstract

**Background:**

The NHS is facing a workforce crisis. Responses to date have focused on improving recruitment of staff, but less attention has been paid to retention.

**Aim:**

To conduct a rapid review using Rosabeth Moss Kanter’s three Ms model of workforce motivation as a sensitising framework to examine the current medical workforce crisis. The work considers how insights from research in other professions offers new thinking for understanding what motivates doctors to continue working.

**Design & setting:**

Rapid literature review with secondary analysis of existing research examining reasons for leaving medicine.

**Method:**

A systematic search strategy was developed with the aid of an information specialist. The search terms used were: medical professionals, retention, and NHS. The exclusions were: commentaries, non-medical professionals, non-English language, and it was limited to post-1990. The search was applied to three electronic databases, MEDLINE, Embase, and Healthcare Management Information Consortium (HMIC). This produced a dataset describing study design, and factors related to motivation for leaving the medical profession. Comparative thematic analysis distilled core themes explaining the reasons for leaving and their relation to the three Ms model.

**Results:**

Of 3389 abstracts identified, screening and assessment produced 82 articles included in the final analysis. Thematic analysis identified four key themes: low morale, disconnect, unmanageable change, and lack of personal and professional support. The themes of mastery, membership, and meaning were substantially present within the dataset.

**Conclusion:**

Kanter's three Ms model of motivation can be applied to the medical workforce to understand retention issues. This work supports the development of targeted solutions to tackle the worsening workforce crisis.

## How this fits in

This study offers new insights into the important and time-critical problem of the medical workforce crisis and the challenge of improving staff retention. Current initiatives focus on extending training numbers and providing financial incentives. This analysis highlights why these initiatives alone may not succeed. Future work should pay attention to understanding and addressing factors in the workplace that can undermine professionals’ sense of worth and value, and the ability to exercise their distinct expertise within a broader community of practice.

## Introduction

The NHS is dealing with a workforce crisis. As was forecast by the King's Fund in 2019,^1^ critical shortages are now being seen within some specialties (general practice, psychiatry), disciplines (nursing, notably community nursing), and geographical locations.^2^ The immediate progression of doctors into specialty training after the foundation programme has fallen from 71.3% to 34.9% (2011–2019).^3^ With more than 100 000 NHS staff vacancies, this crisis poses a bigger threat to the NHS than underfunding.^1^ Recent events, such as Brexit and the COVID-19 pandemic, further highlight the need for a strong healthcare workforce to deliver high quality and effective health care.^4^ This study starts by focusing on the medical profession.

Measures to tackle doctor shortages have focused on recruiting more staff: expanding both undergraduate and postgraduate training places,^5^ and offering golden handshakes to GP trainees taking up posts in the hardest-to-recruit geographical areas.^6^ Less work has been done to understand and address retention issues, with some exceptions; for example, tackling the pension issues contributing to early retirement.^7^

Marchand and Peckham's^8^ 2017 review of the workforce evidence described an emphasis on short-term policies responding to immediate needs. They noted that intrinsic factors (for example, career opportunities and job satisfaction) are more important than extrinsic factors (for example, golden handshakes) in influencing retention.^8^ Importantly, the review highlighted an overall lack of evidence to inform practice and policy.

Outside of the medical profession, there is rich literature describing how to motivate and retain employees.^9^ Based on a distillation of research across a range of professions, Kanter identified three key factors needed to motivate professionals in the workplace: meaning, membership, and mastery (Table 1). All are grounded within the intrinsic motivators flagged by Marchand and Peckham.^8^ Kanter has used this model to understand and address workforce problems within a wide variety of businesses.^10^ To the authors' knowledge, this model hasn’t been applied to the medical profession.

**Table 1.** Three factors described by Kanter as driving motivation of a workforce^9^

	Definition	Explanation
Meaning	Repeat and reinforce a larger purpose	People are motivated to the tasks of daily work by their perceptions of the meaning behind their work — why it matters. People must be supported to develop and engage with an understanding of why what they do matters.If the positive impact of work done by an individual is emphasised regularly, even mundane tasks can become a means to a larger end and so become accepted into everyday actions.
Mastery	Help people develop deep skills	People are motivated to do their job by a desire for mastery — the development and delivery of expertise.When people are given the adequate tools and support for their role, they are better able to complete the tasks with increasing efficiency; even those tasks that may be perceived as routine and mundane.
Membership	Create community by honouring individuality	For people to be motivated to do their job, they must feel part of a community that supports and enables them to flourish both as an individual and collectively.Collective working enables individual strengths and values to be developed and utilised while contributing to broader goals.

This study considered whether Kanter’s model of workforce motivation might help us understand and address the retention crisis within medicine. Two research questions were identified: (1) what factors explain retention problems in the medical profession?; and (2) how do these map to the three Ms model?

## Method

A rapid review was undertaken in order to test the utility of the three Ms model. The aims were:

- to describe factors stated as reasons for leaving the medical profession and the variables that predict and/or explain variation in responses; and
- to analyse whether the 3Ms model can explain current trends in retention.

With the aid of an information specialist (SG), database-specific indexed and text-word terms were used to draft a search strategy for OVID MEDLINE, which was then translated to Embase and HMIC via OVID, based on inclusion and exclusion criteria (Tables 2–3). Preliminary searches identified ‘burnout’ as a key search term to capture the literature on reasons for leaving. The search strategy combined three concepts: medical professionals, AND retention or burnout, AND NHS or United Kingdom (Supplementary Boxes 1–3). Databases were searched from January 1990 to March 2020. Results were loaded into an EndNote library and duplicates removed. As a rapid review,^11^ no additional databases, resources, or supplementary search methods were used.

**Table 2.** Study inclusion criteria

**Inclusion criteria**
Types of studies	Publication date January 1990–March 2020 (selected owing to introduction of the new GP contract and the Calman report that led to pre-modernising medical careers).
	UK only (Northern Ireland, Scotland, England, Wales, Channel Islands).
	English language only.
	Studies using qualitative, quantitative methods, empirical studies, interviews, systematic reviews, and original research.This includes mixed-methods studies.
Types of participants	Medical doctors including junior trainees, specialists, GPs.Mixed group of participants, for example, nurses and doctors only if results from doctors are explicitly separate from other participants.
	Practising in the NHS.
Types of outcome measures	Intrinsic personal motivations; for example, personal attitudes, resilience, coping strategies, work–life balance.
	Workforce, for example, job satisfaction, recruitment and attrition, financial incentives, early retirement, leaving.
	Burden on health professionals.

**Table 3.** Study exclusion criteria

**Exclusion criteria**
Types of studies	Non-English language.
	Published pre-1990.
	Grey literature or not published in a peer reviewed journal.
	Dissertations or theses.
	Proceedings.
	Commentary articles, written to convey opinion or stimulate research and/or discussion, with no research component.
Types of participants	Non-medical healthcare professional, allied healthcare professionals only.
	Dentists, dental practitioners, vets, nurses, medical students.
Types of outcome measures	Anything except doctors’ perspective.
	Switching between specialties.
	Burden on patients, societal perspectives.
	Economic burden at a society level; for example, costs to government or councils.

Titles and abstracts of each result were screened for eligibility by EA with a random 25% double-screened by SG. The second stage of screening used full-text. Data extraction used the headings described in Table 4. Included studies were each extracted by one of five of the authors (EA, KH, MS, BN, SG), with 10% double-reviewed; no disagreements arose.

**Table 4.** Data extraction criteria

Study identifier	Title of article, authors, year of publication, journal name.
Data generation	Location, study method, interface — primary or secondary care, participants.
Study outcomes	The main themes and findings of each article were extracted to aid in explaining reasons for leaving medicine and whether the three Ms model can explain these trends. In extracting information from the dataset, the researchers sought to identify described reasons for leaving the profession. Researchers were sensitised to the three Ms framework proposed by Kanter, remaining open to the possibility of other issues outside of the framework arising.

The three Ms model was used as a framework to sensitise the analysis of the dataset meaning that data were specifically sought out that described, explained, or refuted the three Ms model. However, the authors also remained open to other explanatory themes emerging from the dataset.

Thematic analysis using the constant comparison approach^12^ generated key explanatory themes across the dataset that explained retention issues. This was carried out by EA and JR, with disagreements resolved through discussion. Cross-comparison between emerging descriptive themes and the full data, with particular attention to areas of dissonance and similarity, allowed the researchers to systematically identify core explanatory themes. Details of study design and findings were used to critically explain any differences in explanatory themes identified.

The analysis produced core themes from the dataset describing reasons for retention problems in the medical profession. The discussion considers how these related to the principles of the three Ms.

## Results

**Figure 1.**
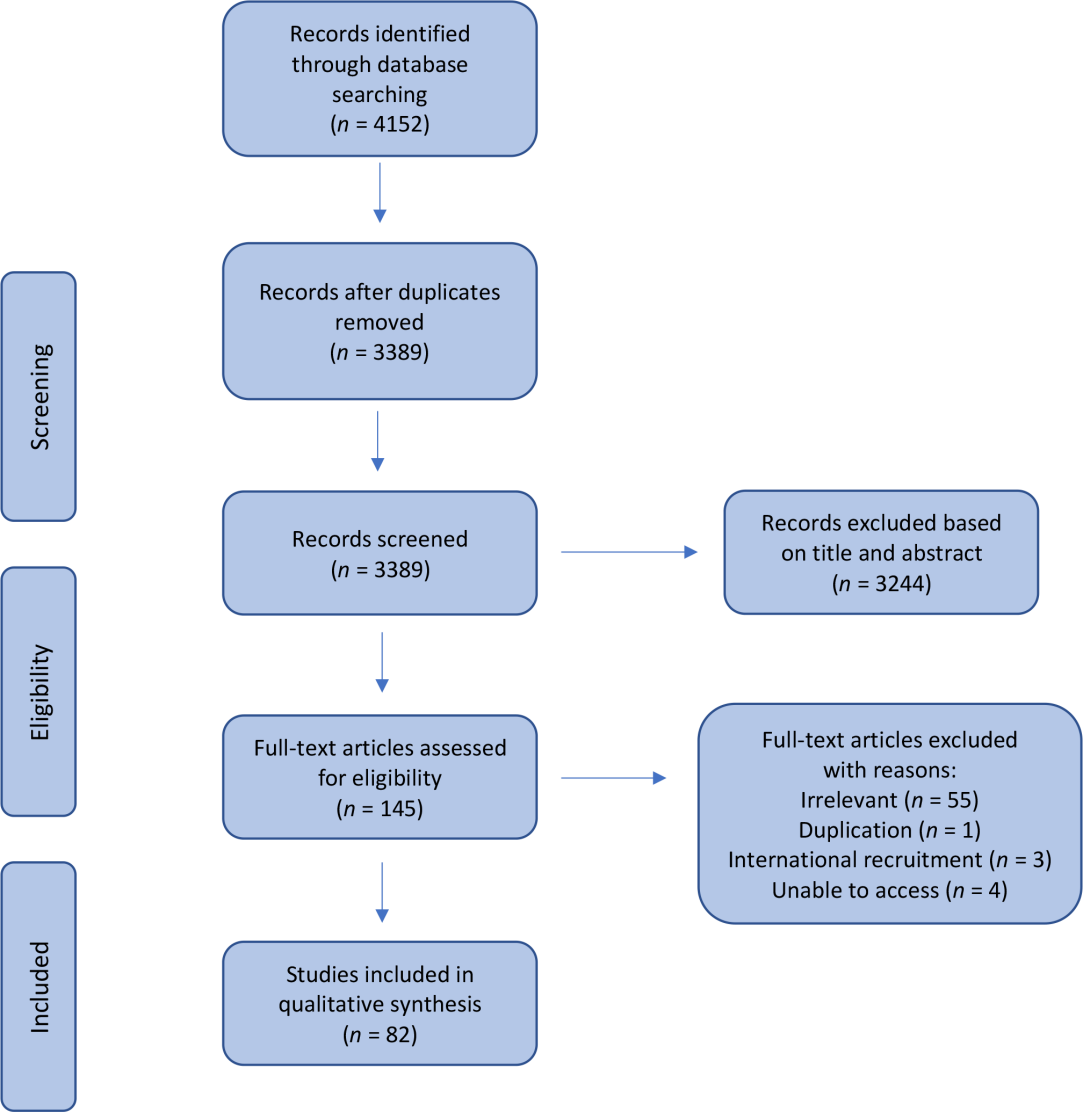
Flow diagram of study selection process

The results of the search are summarised in a PRISMA diagram (Figure 1). The 82 final articles included staff from primary care (*n* = 47),^6,8,13–57^ secondary care settings (*n* = 14),^58–71^ or both (*n* = 21).^72–92^ Studies used online or postal questionnaires (*n* = 59), interviews or focus groups (*n* = 18), systematic reviews (*n* = 2), and a mix of these methods (*n* = 3) to collect data, carrying out qualitative and quantitative analysis.

Thematic analysis of the final included dataset identified four core themes explaining retention problems:

1. Low morale
2. Disconnect
3. Unmanageable change
4. Lack of personal and professional support

### Low morale

Low morale is expressed repeatedly across the dataset. Factors that contribute to low morale include loss of continuity in patient care,^45^ loss of autonomy in clinical practice,^65^ high levels of burnout,^78^ low levels of job satisfaction,^74^ along with increased workload, long working hours, and lack of resources.^18^ Analysis thus revealed the negative impact on doctors of doing an almost undoable job (mastery) on their own sense of purpose (meaning),^44^ leading to burnout. These effects may be compounded by data that reported clinicians feeling undervalued by the health system.^45^

### Disconnect

Evidence across the dataset demonstrated a mismatch between doctor and patient expectations contributing to doctors’ intentions to leave the profession. Professionals described concerns about differences between patient and professional understanding of what was possible, citing high patient expectations; dealing with difficult patients; and fear of complaints or litigation as causes of stress and job dissatisfaction.^17,46^ Perceptions that the public were more demanding and less respectful of healthcare professions^46^ were potentially compounded by negative portrayal of the profession in the media.^17^ Disconnect was also seen at the level of health systems, with Dale *et al* ^20^ highlighting growth in patient expectations unmatched by governmental resource provision contributing to retention issues. Disconnect thus served to undermine meaning and mastery as motivating factors for the profession, but also membership of a shared community between professionals and the public.

### Unmanageable change

While evolving demography and epidemiology make change inevitable, two factors were identified that were perceived to make this unmanageable: inadequate resource, and lack of control.

The impact of discrepancies between need and resources was evident across the dataset. Examples included services being moved into primary care without the equivalent additional supportive resources,^17^ and a failure to increase the availability or duration of appointments to meet the needs of an ageing population with complex needs.^17,22,44^ Dale *et al* ^20^ reported increasing administration and bureaucracy not allowing for the pursuit of other professional interests, which contributed to retention issues within the GP workforce. At a service level, Lester *et al* ^32^ described continual restructuring and uncertainty about the future, as well as increased administrative work and decreased time with patients. Unmanageable change undermines the exercise of mastery, but also disrupts membership of a community.

Analysis also revealed the impact of undermining professionals’ sense of control on retention. Doctors described changing job role, often without prior consultation, as a factor behind reasons to quit. Rizan *et al* ^68^ highlighted regaining control as a reason for F2 doctors taking time out of training before deciding whether to pursue specialty training. Upton *et al* ^71^ reported stress and burnout resulting from loss of control, with no direct relation to workload. Doctors reported concerns about increasing pressures on the NHS with regular restructuring and underfunding resulting in an inability to provide high quality health care as reported in Sansom *et al*.^44^ Unmanageable change also impacts on meaning as a motivating factor for the medical workforce.

### Lack of personal and professional support

Lack of support at a personal and professional level for doctors was highlighted across the dataset, with increasing levels of burnout and poor mental health among doctors on the rise.^34,41,51^ Detrimental stress, owing to the demands and bureaucracy involved with revalidation, appraisals, and exams, compounded by a lack of support, was reported by Dale *et al*.^19^ Doctors reported lower levels of perceived support from NHS management compared with those outside of the NHS or abroad.^90^ Lack of supervision and mentorship, both during clinical practice and with career progression contributed to trainees feeling unsupported.^64^ Gregory *et al* ^77^ reported improved job satisfaction outcomes associated with interventions to address supervision and mentorship.

These effects are not equally distributed across the workforce. Sibbald *et al* ^46^ highlighted men reported less job satisfaction than women, although women were shown to have more mental health symptoms as seen in Newbury-Birch *et al*.^66^ Poor work–life balance and the demands of family commitments has also been shown to affect women more than men.^31^ Lower job satisfaction in doctors from minority ethnic backgrounds and those serving urban and deprived populations is also observed.^46^ Secondary care doctors self-reported high levels of job satisfaction in Sharma *et al*'s study,^69^ despite high levels of depersonalisation, emotional exhaustion, and burnout.

The theme of personal and professional support was seen to map to Kanter's third category of membership, which is the importance of building communities of practice, which support and enable individuals to thrive in their role and provides the motivation to stay.

## Discussion

### Summary

The analysis described four key themes explaining reasons for leaving medical practice: low morale, disconnect, unmanageable change, and lack of support. The findings resonate with Marchand and Peckham's^8^ review in highlighting the importance of intrinsic factors, such as job satisfaction linked to workload, in explaining retention of doctors. Disconnect, unmanageable change, and lack of personal and professional support have been highlighted as additional elements. Reflection within the team considered how the themes arising related to Kanter’s model of motivation (Table 1).

The following now considers how Kanter’s work may offer new insights into how to motivate the medical workforce.

#### Meaning

Meaning is an important motivating factor in a workforce (Table 1), needed to enable both the mundane everyday tasks to continue as well as individuals to thrive.^9,10^ The analysis highlighted the impact of both unmanageable change and disconnect in undermining professionals’ sense of meaning and purpose in their work. At a time of high workload and pressures, professionals described uncertainty in whether their work is valued by both the public and the ‘health system’. The authors described how a low sense of meaning may be contributing to the low morale that leads to burnout and leaving the profession. Applying Kanter’s model to the analysis, the need to pay attention to reviewing and revitalising professionals’ sense of meaning in a rapidly changing health service context is highlighted if the retention crisis is to be addressed. The challenges of recovering from COVID-19 underline the urgency of this work.

#### Mastery

For a workforce to remain motivated, the workplace must offer opportunities to both utilise existing expertise, and extend and develop their role. The analysis highlighted several themes that undermine the exercise and development of mastery within the medical profession. Unmanageable change described a rapidly shifting service context that makes it increasingly difficult for doctors to exercise their current expertise, let alone extend and develop new areas such as portfolio careers.^20^ Doctors felt unconfident in using their professional expertise for fear of missing the expectations^93^ of patients or health systems. The findings highlight a need to review and address the contextual factors that undermine the exercise of mastery (including pressure of workload and performance management targets) to both motivate those still working within the profession and stem the tide of those leaving.

#### Membership

Kanter described why it is important to attend to an understanding of community in addressing workforce motivation. The themes of disconnect and unmanageable change highlight the significance of the speed and scale of change in the NHS in undermining a sense of community. This has been compounded by disruptions to personal and professional support. Reorganisation has been a regular feature in the NHS for some years, acutely compounded by the challenges of COVID-19. The focus to date has been on a goal of integration, smoothing the pathway for a patient through the service. A multitude of initiatives have been seen including development of new clinical roles, new networks, and the movement of work between primary and secondary care settings. While integration remains an important goal, the analysis in this study and Kanter’s work are reminders that attention must also be paid to the impact on professional networks and sense of membership.

The findings, therefore, suggest that addressing the retention crisis in the medical profession will need three distinct elements not currently addressed by workforce initiatives. COVID-19 recovery plans may provide an opportunity for new conversations between the profession, the public, and policymakers on the purpose of the NHS: revisiting and revitalising shared expectations of what it can and can’t do (meaning and value); and the distinct value of the medical profession in a wider multidisciplinary workforce (mastery and membership).

The scope to translate these ‘higher level’ discussions into actions at practice level is also recognised. Kanter^10^ offers rich descriptions of using the three Ms model to make active improvements to workforce motivation in a number of case studies. Although much of her work is in the private sector, the principles could be translated into the NHS context. Initiatives included: deprioritising working weeks to enable employees to undertake (lead) their own quality improvement projects; buddying and mentoring programmes, which also support outside-of-work activities and broader personal development; and active engagement during work time with community service activities.

### Strengths and limitations

This rapid review is a timely contribution to the factors affecting the current NHS workforce crisis. It introduces the three Ms themes of motivating a workforce: meaning, mastery, and membership. The review highlights how these themes might be utilised to aid in further discussions aimed at effectively tackling recruitment and retention in the NHS. The use of a systematic search and theory-informed analysis, as well as its resonance with the results of the Marchand and Peckham^8^ review, are strengths of this article.

Limitations included a narrow database search (Embase, MEDLINE, and HMIC) owing to the time constraint on completing this piece of work. Most of the included studies were questionnaires or surveys with no clear validation tools highlighted in some of the articles, possibly introducing a risk of bias. The intention of this work was to rapidly, critically, and transparently consider the potential for further, more detailed work.

### Comparison with existing literature

Current literature such as Owen *et al*,^35^ Sansom *et al*,^45^ Hann *et al*,^27^ Dale *et al*,^20^ have rightly focused on recruitment and retention issues in the workforce, with the present study's findings similar to those of the Marchand and Peckham^8^ review. This review is the first to examine whether the three Ms model proposed by Kanter in non-healthcare settings can help in the understanding and effective tackling of the current NHS workforce crisis hence, existing literature on this topic is not yet available.

### Implications for research and practice

The worsening workforce crisis in the NHS is an issue that needs addressing urgently. Current strategies are not tackling this effectively with more healthcare professionals still leaving the NHS.

The current strategies to tackle the workforce crisis focus on hiring more staff, expanding the number of GPs in training, with a target of 15 000 new GPs between 2015 and 2020, as well as adding 1500 new places to medical schools.^5^ Yet the analysis shows that low job morale, disconnect, lack of personal and professional support, and unmanageable change all contribute to demotivating a workforce.

New strategies need to be developed to continually provide a high quality, safe, and effective NHS for all. Future work should pay attention to understanding and addressing barriers to the workplace supporting professionals’ sense of worth and value, and their ability to exercise their distinct expertise within a broader community of practice. This analysis provides support for the use of the three Ms model in redesigning policy and practice in this area.

New research is now needed to examine the enablers and barriers to development of each element of meaning, mastery, and membership in the current workplace. Kanter’s model provides an evidence-based framework to support the systematic development and evaluation of retention interventions. Within the general practice setting, the present authors propose the need for multiprofessional and patient input into systematically describing what changes are needed, and developing, implementing and evaluating their effects. Drawing on Kanter’s experience,^10^ the authors further propose that this work needs to be ‘bottom-up’, led by — and so supporting the development of — communities of practice on the ground.
